# Epilepsy-Linked Gut Microbiota and Metabolic Signatures in Acquired Epilepsy: The Focus on Short-Chain Fatty Acid and Tryptophan Metabolism

**DOI:** 10.3390/biom16081098

**Published:** 2026-07-27

**Authors:** Teresa Ravizza, Rossella Di Sapia, Akash Bera, Claudia Fracasso, Jacopo Lucchetti, Marco Gobbi, Annamaria Vezzani

**Affiliations:** 1 Laboratory of Epilepsy and Therapeutic Strategies, Department of Acute Brain and Cardiovascular Injury, Istituto di Ricerche Farmacologiche Mario Negri IRCCS, 20156 Milano, Italy; teresa.ravizza@marionegri.it (T.R.); rossella.disapia@marionegri.it (R.D.S.); akash.bera@marionegri.it (A.B.); 2 Department of Biochemistry and Molecular Pharmacology, Istituto di Ricerche Farmacologiche Mario Negri IRCCS, 20156 Milano, Italy; claudia.fracasso@marionegri.it (C.F.); jacopo.lucchetti@marionegri.it (J.L.); marco.gobbi@marionegri.it (M.G.)

**Keywords:** drug-resistance, gut dysbiosis, short-chain fatty acids, kynurenines

## Abstract

Epilepsy is increasingly recognized as a systemic disorder involving complex interactions between the brain and peripheral systems. Among these, the gut microbiota has emerged as a key regulator of host metabolism and immune homeostasis through the production of bioactive metabolites that mediate the communication between gut and brain. In recent years, growing evidence has linked gut dysbiosis to epilepsy, particularly in drug-resistant forms, and interventional studies targeting the gut microbiota in animal models suggest that microbiota-driven metabolic alterations may contribute to seizure generation and recurrence, as well as the associated neuropathology and cognitive deficits. In this review, we summarize current knowledge on the role of the gut microbiota–metabolome axis in acquired epilepsy, with a particular focus on short-chain fatty acids (SCFAs) and tryptophan-derived pathways. SCFAs represent major microbial products involved in energy metabolism, inflammation, blood–brain barrier integrity, neurotransmission and epigenetic mechanisms. In parallel, microbiota-dependent tryptophan metabolism represents a central hub linking intestinal microbial activity to brain function through serotonin, kynurenine, and indole pathways. Dysregulation of these pathways may influence neuronal excitability and contribute to seizures. Converging evidence supports the concept that epilepsy is associated with a coordinated alteration of gut microbial composition and host–microbiota metabolic interactions. However, further research is needed to elucidate the mutual communication between the gut and its microbiota and the metabolic flux, and their influence on brain function in neurological conditions. A better understanding of the underlying pathways and mechanisms may highlight novel therapeutic strategies and discover novel biomarkers of disease trajectory.

## 1. Introduction

The mammalian gastrointestinal tract harbors a highly diverse microbial ecosystem that contributes to multiple aspects of host physiology, extending beyond digestion and nutrient metabolism. Through mutual interactions, the gut microbiota has emerged as a major regulator of brain function and behavior [1]. This concept is embodied in the gut–brain axis, a bidirectional communication network involving neural, immune, endocrine, and metabolic pathways that enables reciprocal signaling between the gastrointestinal tract and the central nervous system (CNS) [1].

Increasing evidence indicates that disruption of gut microbial homeostasis, commonly referred to as dysbiosis, is associated with various neurological disorders, including epilepsy [2,3]. In fact, variations in microbiota composition have been linked to stress, anxiety, and major depressive and neuropsychiatric disorders [2]. In the context of epilepsy, both clinical and experimental studies have identified disease-associated microbial and metabolic signatures, some of which have been linked to seizure burden, cognitive dysfunction, and resistance to antiseizure medications [3,4]. Alterations in microbial composition and metabolic activity may affect neuroinflammatory responses and blood–brain barrier integrity, thereby promoting neuronal excitability and influencing seizure susceptibility and epilepsy progression [1,3]. Given that approximately one-third of people with established epilepsy remain refractory to currently available therapies [5], and interventions halting disease progression are lacking, there is growing interest in identifying novel biological mechanisms that could serve as targets for disease-modification approaches that affect mechanisms underlying epileptogenesis and the associated neurological comorbidities.

Among the pathways connecting the gut microbiota to brain function, microbial metabolites have emerged as key mediators. In particular, lipid mediators and tryptophan metabolites may regulate immune responses, cellular metabolism, epigenetic mechanisms, and neuroglial functions which are implicated in epilepsy [3].

In this review, we summarize current evidence linking gut microbiota alterations and metabolic dysfunction to epilepsy, with a particular focus on microbiota-derived metabolites and their potential role as biomarkers and therapeutic targets.

## 2. Systemic Alterations in Epilepsy: Implications for Gut–Brain Axis Dysfunction

Epilepsy is a brain disease characterized by enduring predisposition to generate epileptic seizures and by neurological, cognitive and psychological consequences [6]. It represents the third leading contributor to the global burden of disease for neurological disorders and affects approximately 51.7 million people worldwide [7].

Epilepsy has traditionally been regarded as a disorder of the CNS; however, it is increasingly recognized as a complex systemic condition that affects multiple physiological systems beyond the brain. In addition to its defining feature—the predisposition of the brain to generate recurrent unprovoked seizures—epilepsy is associated with a broad spectrum of somatic comorbidities and dysfunctions involving peripheral organs and tissues [8]. Emerging evidence suggests that common pathophysiological mechanisms, including chronic inflammation, oxidative stress, immune dysregulation, and metabolic disturbances, contribute not only to epileptogenesis—the process through which a normal brain undergoes molecular, cellular, and network alterations that ultimately lead to the development of epilepsy [9]—but also to the systemic manifestations of the disease. Among the most frequently reported comorbidities are cardiovascular diseases, respiratory disorders, metabolic abnormalities, and systemic autoimmune conditions [8,10]. Gastrointestinal disturbances are also highly prevalent in individuals with epilepsy [11], further supporting the concept that epilepsy extends beyond a brain-centered disorder and involves complex interactions between the CNS and peripheral physiological networks.

### 2.1. The Gut–Brain Axis in Epilepsy

The human gastrointestinal tract hosts a complex and dynamic microbial ecosystem that contributes to numerous physiological processes, including nutrient metabolism, immune maturation, maintenance of epithelial barrier integrity, and regulation of host metabolic homeostasis. Beyond its local functions, the gut microbiota is increasingly recognized as an important modulator of brain physiology through a bidirectional communication network commonly referred to as the gut–brain axis [1]. This network integrates neural, endocrine, immune, and metabolic signaling pathways that enable continuous communication between the gastrointestinal tract and the CNS. Key components of this axis include the enteric and autonomic nervous systems, vagal afferents, neuroendocrine pathways, immune mediators, and a broad range of microbially derived metabolites capable of reaching peripheral tissues and the brain. Through these interconnected mechanisms, gut microorganisms can influence neuronal activity, neuroinflammatory responses, blood–brain barrier function, and synaptic plasticity [1].

Experimental studies have provided compelling evidence that the gut microbiota is involved in neurodevelopment and brain function. Animals raised under germ-free conditions exhibit abnormalities in neural signaling, altered glial cell activity, increased blood–brain barrier permeability, and behavioral deficits affecting cognition, emotional processing, and stress responses. These observations support the concept that microbial communities contribute to the maintenance of CNS homeostasis throughout life [1,12].

In recent years, alterations in gut microbial composition and function have been increasingly associated with epilepsy. Distinct microbial signatures have been reported in both adult and pediatric patients, particularly in individuals with drug-resistant epilepsy [13,14], and similar alterations have been reproduced in several experimental models of acquired and genetic epilepsies [15,16,17,18,19]. Importantly, studies employing microbiota-transfer approaches have demonstrated that transplantation of dysbiotic microbial communities can increase seizure susceptibility in recipient animals, whereas transfer of microbiota from healthy donors may exert protective effects and reduce seizure burden [19,20,21,22,23]. Additional support for a causal role of the microbiota comes from dietary interventions, including ketogenic diets and probiotic-based approaches, which have been associated with both microbial remodeling and improved seizure control [24,25].

Although the specific mechanisms linking gut dysbiosis to epileptogenesis remain incompletely understood, growing evidence suggests that alterations in microbial metabolic activity may represent a critical interface between the gut and the brain. Changes in the production of microbiota-derived metabolites can influence neuroinflammatory pathways, oxidative stress responses, neurotransmitter systems and cellular energy metabolism, all of which have been implicated in epilepsy pathophysiology [26,27,28]. Consequently, increasing attention has been directed toward identifying epilepsy-associated metabolic signatures and elucidating how microbiota-derived metabolites contribute to disease progression and treatment response.

### 2.2. Metabolic Signatures of Gut Dysbiosis in Epilepsy

Recent untargeted metabolomics studies in both individuals with focal epilepsy and related experimental models have revealed profound alterations in metabolic pathways associated with epilepsy, particularly involving lipid metabolism, amino acid turnover, and cellular energy homeostasis [16,17,27,29].

Evidence from a preclinical model of acquired epilepsy in which seizures develop after an acute brain injury caused by status epilepticus and epilepsy occurs in approximately 50% of animals, provided a unique experimental setting. This model enabled comparison of gut morphology, microbial composition and systemic metabolic profiles among animals that developed epilepsy, animals exposed to the same insult but remaining seizure-free and sham controls. Notably, epilepsy development was associated with marked alterations in intestinal structure and function. Epileptic animals exhibited a reduced villus height-to-crypt depth ratio and decreased numbers of goblet cells in the duodenum, suggesting impaired epithelial homeostasis and reduced absorptive capacity. These changes were accompanied by increased macrophage infiltration and elevated markers of inflammation and oxidative stress, indicating the presence of a persistent inflammatory state within the intestinal microenvironment [16].

Metagenomic profiling of feces further revealed epilepsy-associated alterations in microbial communities, including changes in bacterial taxa known to participate in the production of SCFAs [16]. The selective reduction in SCFA-producing microorganisms identified a microbial signature associated with epilepsy and suggested a disruption of microbiota-derived metabolic functions. Consistent with these microbial alterations, epileptic animals displayed a distinct systemic metabolic profile characterized predominantly by dysregulation of lipid-related pathways. Among the most prominent changes were alterations in linoleic acid metabolism and the biosynthesis of unsaturated fatty acids, together with pathways involving glycine, serine, and threonine metabolism [16]. The observed increase in linoleic acid-related metabolism is of particular interest because this omega-6 polyunsaturated fatty acid serves as a precursor of arachidonic acid and downstream prostanoids, which have been implicated in neuroinflammation and oxidative stress known to contribute to epileptogenesis [26].

Although metagenomic analyses have substantially expanded our understanding of gut microbial composition in health and disease, taxonomic profiling alone provides limited insight into the functional consequences of microbiome alterations. Increasing evidence indicates that the gut microbiota exerts many of its effects on the host through the production and biotransformation of a wide range of metabolites, including SCFAs, bile acids, amino acid derivatives, and neurotransmitter-related compounds. Consequently, the host metabolome can be viewed as an integrated functional readout of host–microbiome interactions, reflecting both microbial metabolic activity and host responses [30]. The integration of fecal metagenomics with metabolomic profiling therefore represents a powerful approach to move beyond descriptive associations and uncover mechanistic pathways linking gut dysbiosis to disease pathogenesis.

To investigate the functional relationship between microbial alterations and host metabolism, microbial pathway predictions derived from metagenomic analyses were compared with circulating metabolomic profiles in epileptic animals [16]. Interestingly, only a limited correspondence was observed between predicted microbial metabolic functions and blood metabolite alterations. This finding likely reflects the fact that circulating metabolites integrate contributions from multiple host organs and physiological processes in addition to the gut microbiota. Consequently, fecal metabolomic profiling may provide a more direct assessment of microbiota-derived metabolic activity and could represent a valuable complementary approach for characterizing functional consequences of gut dysbiosis in epilepsy.

**Figure 1:**
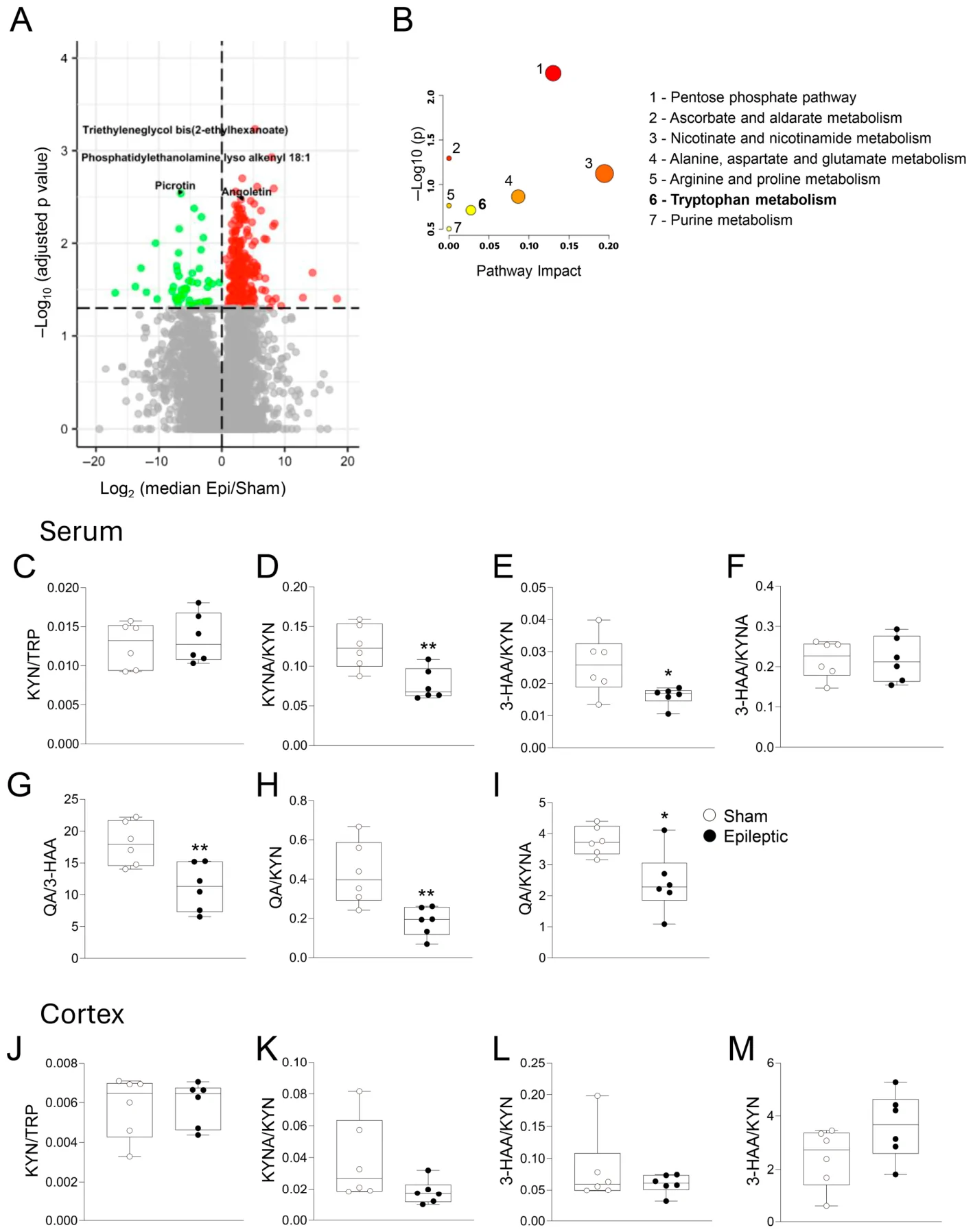
Metabolomic profile in epileptic mice vs. sham controls. (**A**) Volcano plot illustrates metabolites differentially abundant in feces of epileptic mice vs. sham controls. Analyses were conducted using mean metabolite abundance values with an adjusted *p*-value threshold of *p* < 0.05. Metabolites that decreased or increased in epileptic mice (Epi) compared to Sham (healthy controls) are shown in green and red, respectively. (**B**) Pathway enrichment analysis identifies statistically significant up-regulated metabolic pathways in feces. Each dot represents a metabolic pathway; dot size reflects pathway impact (metabolite centrality), while color intensity (red-to-yellow) depicts enrichment significance (higher-to-lower *p*-values). Dot numbers correspond to the pathways shown on the right, ordered by their impact (from high-to-low centrality). An insufficient number of significantly altered metabolites to support pathway analysis relates to downregulated pathways in the feces, and in the corresponding serum samples [17]. Data are extracted from [17] and reproduced with permission from John Wiley and Sons. (**C**–**M**) Assessment of kynurenine metabolite ratios in serum (**C**–**I**) and cortex (**J**–**M**) of epileptic mice and sham controls (*n* = 6/group) [43]. These molecules were quantified using an updated and fully validated UHPLC–MS/MS method [44]. * *p* < 0.05, ** *p* < 0.01 by unpaired *t*-test.

Based on this evidence, a subsequent study analyzed both fecal and blood metabolomic profiles in a mouse model of status epilepticus-induced acquired epilepsy [17], revealing gut dysbiosis that partially overlapped with that previously reported in the rat model. In contrast to the limited metabolic alterations detected in circulation, fecal metabolomic analyses in epileptic mice revealed a broader and more pronounced remodeling of metabolic pathways [17]. Notably, several pathways involved in cellular redox regulation, energy metabolism, amino acid turnover, and nucleotide biosynthesis were selectively altered within the intestinal compartment of epileptic mice compared with control mice. These included the pentose phosphate pathway, ascorbate and aldarate metabolism, nicotinate and nicotinamide metabolism, alanine–aspartate–glutamate metabolism, arginine and proline metabolism, purine metabolism, and tryptophan metabolism (Figure 1A,B) [17]. Such changes are likely to reflect shifts in microbial metabolic activity as well as altered host–microbiota interactions affecting antioxidant defenses, energy utilization, and amino acid metabolism. The preferential detection of these metabolic alterations in fecal samples, together with their absence in serum, suggests that microbiota-associated metabolic changes may remain compartmentalized within the intestinal lumen and therefore may not be fully captured by systemic metabolomic analyses. Accordingly, fecal metabolomics may complement circulating metabolomic analyses by providing additional information on microbiota-associated functional alterations that are not reflected in plasma, and may therefore represent a valuable approach for identifying metabolic signatures linked to epilepsy.

In summary, these preclinical findings in animal models of acquired epilepsy support the existence of coordinated alterations involving intestinal integrity, microbial composition and metabolism, highlighting that the gut microbiota–metabolome axis is a potential source of biomarkers and therapeutic targets [18].

### 2.3. Short-Chain Fatty Acids: Linking Gut Dysbiosis to Epilepsy

While taxonomic and metabolomic studies consistently support the presence of gut ecosystem alterations in epilepsy, the molecular mediators through which these changes influence brain function remain incompletely understood. Among the candidate metabolites, SCFAs have emerged as pivotal signaling molecules at the microbiota–gut–brain interface, prompting investigation of their dysregulation in both experimental and clinical studies [16,17,31,32].

SCFAs, primarily acetate, propionate, and butyrate, are among the most extensively studied microbiota-derived metabolites owing to their broad effects on host physiology. Generated through the fermentation of dietary fibers by gut bacteria, SCFAs participate in multiple processes relevant to brain health, including energy homeostasis, maintenance of blood–brain barrier function, regulation of neuroimmune responses, modulation of glial activity, and neurotransmitter signaling, which are all processes implicated in epileptogenesis and the consequent seizure generation [26]. Their biological actions are mediated through the activation of free fatty acid receptors as well as epigenetic mechanisms, notably the inhibition of histone deacetylases, which can influence gene expression programs involved in neuronal plasticity and inflammation [32]. Accordingly, alterations in SCFA production and signaling have been reported in both preclinical models of epilepsy and clinical studies [16,17,31].

In particular, we recently reported a reduced abundance of taxa associated with SCFA production in a mouse model of acquired epilepsy compared to healthy mice [17], and chronic supplementation with a balanced SCFA mixture reduced seizure progression, cognitive deficits, and restored gut–brain integrity in this epilepsy model. These functional outcomes were accompanied by neuroprotective effects and reduced neuroinflammation in the hippocampus, as well as normalization of gut structure and cellular composition [17]. Furthermore, we found that SCFA treatment normalized the reduced cortical levels of acetate and enhanced propionate levels in epileptic mice. Acetate, propionate, and related microbial metabolites can serve as alternative metabolic fuels for both neurons and astrocytes, supporting mitochondrial ATP generation and helping maintain ionic gradients during periods of increased neuronal activity, such as during seizures, therefore preserving cell viability. By contributing carbon substrates to the tricarboxylic acid cycle, these metabolites may also sustain intermediary metabolism and favor GABA biosynthesis, thereby promoting a more balanced excitatory–inhibitory neurotransmission profile [33,34]. Notably, acetate and propionate can attenuate astrocyte activation and preserve their key homeostatic functions such as glutamate clearance, glutamine recycling, and water balance regulation, and reduce neuroinflammatory and oxidative stress responses [35,36]. These actions may contribute to the stabilization of neuronal networks and synaptic activity [26].

Through the convergence of metabolic, neuroimmune, and epigenetic effects, SCFAs have emerged as plausible mediators of microbiota-driven influence on epileptogenesis [17]. Consequently, strategies aimed at restoring SCFA-producing microbial communities or enhancing SCFA signaling pathways are being explored as potential therapeutic approaches for epilepsy [37].

Integrated analyses of fecal and circulating metabolites in mice developing epilepsy suggest that many of the metabolic effects elicited by SCFAs originate within the intestinal environment and subsequently may extend to systemic pathways [17]. The more pronounced metabolic remodeling observed in the gut supports the view that SCFAs primarily reshape local microbial–host metabolic interactions, influencing pathways involved in energy production, amino acid turnover, and cofactor biosynthesis. Among these, vitamin B6-related metabolic pathways have emerged as a particularly relevant pathway, as increases have been detected across both intestinal and blood compartments, highlighting a potential metabolic link between gut-derived signals and brain function [17]. SCFAs may influence vitamin B6-related metabolic pathways indirectly by modulating microbial communities capable of vitamin biosynthesis and by regulating host metabolic processes that interact with vitamin B6/pyridoxal-5-phosphate-dependent enzymatic reactions [38]. Therefore, the increased representation of vitamin B6-related pathways observed following SCFA treatment may reflect broader microbiota-mediated metabolic adaptations rather than a direct effect of SCFAs on vitamin B6 metabolism. Since vitamin B6 serves as an essential cofactor for enzymatic reactions involved in neurotransmitter synthesis, including the production of GABA, serotonin, and catecholamines, modulation of this pathway may represent one mechanism through which SCFAs influence neuronal excitability and neurochemical homeostasis in the brain.

In parallel, epilepsy- and SCFA-related metabolic adaptations extend to tryptophan metabolism, a major source of neuroactive compounds involved in gut–brain communication [39,40]. Interestingly, alterations in tryptophan-derived metabolites appear in the feces of epileptic mice (Figure 1A,B) but no changes in tryptophan metabolism were detected in blood [17]. Differently, SCFA administration in epileptic mice specifically upregulated tryptophan metabolism in blood suggesting that peripheral metabolism may contribute to the neurological effects of SCFAs [17]. Emerging evidence indicates a tight interplay between SCFAs and tryptophan metabolism [39,41,42]. SCFAs can modulate host tryptophan catabolism by influencing serotonin biosynthesis and inflammatory pathways controlling kynurenine production, whereas alterations in SCFA-producing microbial communities are often accompanied by changes in microbiota-derived indole metabolites, collectively shaping gut–brain communication.

### 2.4. Microbiota-Dependent Tryptophan Metabolism

The gut microbial metabolism provides different precursors involved in biosynthesis of neurotransmitters, such as catecholamines (dopamine, noradrenaline, adrenaline), GABA, and glutamic acid. In addition, by contributing to the metabolism of the essential amino acid tryptophan, gut bacteria play a role in the synthesis of neuroactive molecules such as serotonin, kynurenines, and indole compounds, with important physiological roles in the gut–brain axis signaling. Tryptophan metabolism has attracted particular attention because of its dual role in neurotransmission and neuroimmune regulation. By influencing the balance between serotonin production and kynurenine and indole pathways, microbiota-dependent alterations in tryptophan metabolism [1,39,40,41,42,45] may have profound consequences for brain homeostasis and seizure susceptibility [46].

While a small fraction of dietary tryptophan is converted into serotonin or transformed into microbial indole derivatives, most systemic tryptophan is metabolized through the kynurenine (KYN) pathway. The first and rate-limiting step of this pathway is catalyzed by tryptophan-2,3-dioxygenase (TDO), predominantly expressed in the liver, and by indoleamine-2,3-dioxygenase (IDO1 and IDO2), which are distributed in extrahepatic tissues including the intestinal mucosa. Unlike TDO, whose activity largely reflects physiological tryptophan metabolism, IDO enzymes are highly responsive to inflammatory stimuli, making the KYN pathway a key interface between immune activation, gut physiology, and brain function [41,47]. Alterations in tryptophan metabolism have been reported across a range of neurological disorders and are recognized as potential contributors to brain disease pathogenesis [48]. Because circulating tryptophan and some KYN metabolites readily cross the blood–brain barrier, peripheral metabolic changes can influence cerebral KYN metabolism. Indeed, a substantial proportion of brain kynurenines originates outside the CNS, whereas local production is mainly carried out by glial cells [49,50]. Consequently, dysregulation of intestinal or systemic tryptophan metabolism may directly affect the balance of neuroactive metabolites within the brain.

Among KYN pathway metabolites, quinolinic acid (QA) and kynurenic acid (KYNA) have attracted particular attention in epilepsy because they exert largely opposing biological effects [48]. QA, generated predominantly by activated microglia, is a potent neuroactive metabolite with pro-excitatory and neurotoxic properties. In contrast, KYNA, which is mainly synthesized by astrocytes, is generally regarded as a neuroprotective molecule with anticonvulsant potential [50,51]. Experimental studies have demonstrated that intracerebral administration of QA induces seizures and neuronal injury, whereas KYNA counteracts excitotoxic mechanisms and reduces seizure susceptibility in several animal models. The divergent effects of QA and KYNA are in part explained by their actions on glutamatergic neurotransmission [46,47,50]. QA acts as an agonist at N-methyl-D-aspartate receptors, enhances glutamate release, and impairs astrocytic glutamate uptake, thereby promoting neuronal hyperexcitability [52,53]. Conversely, KYNA functions as an endogenous modulator of glutamate signaling through antagonistic actions at ionotropic glutamate receptors. In addition, KYNA interacts with several non-glutamatergic targets, including α7 nicotinic acetylcholine receptors, GPR35, and the aryl hydrocarbon receptor (AhR), extending its regulatory influence on neuronal and immune pathways [54]. Beyond their effects on synaptic transmission, QA and KYNA differentially regulate neuroinflammatory and oxidative stress responses. QA promotes reactive gliosis, oxidative damage, and impairment of astrocytic homeostatic functions whereas KYNA exerts anti-inflammatory and antioxidant effects that may contribute to neuronal protection and network stability [55]. Activation of the KYN pathway during systemic inflammation increases the production of circulating kynurenines, providing a mechanistic link between peripheral immune responses and CNS dysfunction. Although QA and KYNA exhibit limited permeability across an intact blood–brain barrier, pathological conditions associated with blood–brain barrier disruption such as in epilepsy, may facilitate the entry of peripheral kynurenines into the brain, amplifying the impact of systemic tryptophan metabolism on neuronal excitability and epileptogenesis. Based on this evidence, we hypothesized that the gut alterations and inflammation we have observed in epilepsy models [15,16,17] may result in altered tryptophan metabolism and consequently dysregulation of the kynurenine pathway with increased production of QA and reduced KYNA. Whether the peripheral changes in the circulating molecules are mirrored in the brain, this would result in an increased QA/KYNA ratio with pathological consequences, such as increased excitotoxicity and neuroinflammation. In support, an increased QA/KYNA ratio has been measured in human temporal lobe epilepsy specimen vs. autoptic control tissue [56,57].

Serum analysis of kynurenine pathway metabolites in epileptic mice revealed a significant reduction in the flux from kynurenine toward both KYNA and QA (Figure 1C–I) [43], indicating peripheral dysregulation of this pathway. In parallel, metagenomic functional profiling showed an increased fecal tryptophan metabolic activity (Figure 1A,B) [17], suggesting a possible metabolic shift in tryptophan utilization toward serotonin and/or indole pathways. However, direct measurements of serotoninergic and indole-derived metabolites are required to confirm this hypothesis. Interestingly, patients with focal epilepsy showed increased serum concentrations of metabolites related to fatty acids and lower levels of metabolites related to amino acids, including tryptophan and kynurenines, compared with the corresponding controls [27]. In contrast, cortical levels of kynurenine metabolites and their ratios remained unchanged in epileptic mice, indicating a dissociation between peripheral metabolic alterations and brain kynurenine pathway homeostasis under these conditions (Figure 1J–M) [43].

A potential consequence of microbiota-driven alterations in tryptophan metabolism leading to a reduction in the systemic pool of kynurenines may impair the activation of the anti-inflammatory type I interferon (IFN-I)/AhR signaling. Thus, metabolites such as kynurenine, kynurenic acid, and xanthurenic acid can activate AhR signaling. Impaired AhR activation has been linked to enhanced mucosal inflammation, which we have observed in experimental epilepsy models [15,16] while restoration of AhR signaling exerts protective effects in experimental models of colitis and inflammatory bowel disease [58].

### 2.5. Specific Microbial Alterations Associated with SCFA and Tryptophan Metabolism in Epilepsy

Recent systematic reviews indicate that gut microbial dysbiosis in epilepsy is characterized by alterations in several taxa involved in the production of neuroactive metabolites, although considerable heterogeneity exists across studies [13,14,31]. However, several taxa involved in microbial metabolite production appear to be recurrently affected. In particular, reductions in SCFA-producing bacteria, including members of the families *Lachnospiraceae* and *Ruminococcaceae* [59], and genera such as *Roseburia* and *Blautia*, have been described in patients with epilepsy [31] and experimental models of acquired epilepsy [16,19]. Since these bacteria are major producers of butyrate and other SCFAs, their depletion may contribute to impaired intestinal barrier function and altered immune regulation. Likewise, bacterial genera such as *Lactobacillus*, *Bifidobacterium*, *Bacteroides*, and *Clostridium*, which participate in tryptophan metabolism through the production of indole derivatives or modulation of the kynurenine and serotonin pathways [41], have also been reported to be altered in epilepsy [31]. These observations support the hypothesis that gut microbial dysbiosis may contribute to the disturbances in SCFA and tryptophan metabolism observed in epilepsy.

## 3. Conclusions

**Figure 2:**
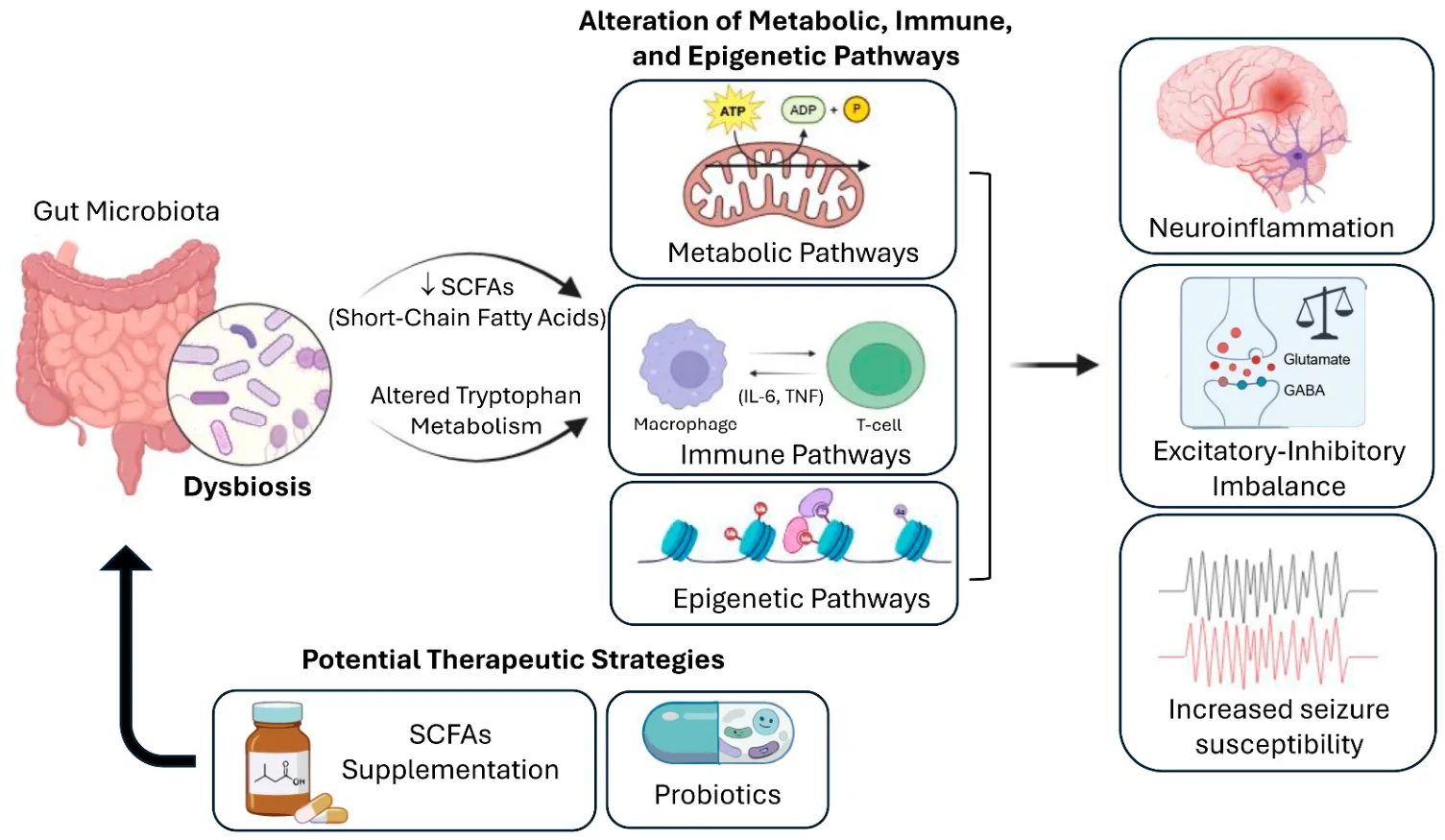
Schematic representation of gut microbiota–metabolome interactions in epilepsy. Dysbiosis is associated with reduced short-chain fatty acid (SCFA) production and altered tryptophan metabolism, leading to altered metabolic, immune, and epigenetic pathways. These changes converge on the brain, promoting neuroinflammation, excitatory–inhibitory imbalance, and seizure susceptibility. Microbiota-targeted interventions, including SCFA supplementation and probiotics, represent potential adjunctive therapeutic strategies to reduce pathological outcomes in epilepsy. See text for details.

This review reports recent evidence supporting that drug-resistant epilepsy is associated with alterations of the gut–microbiota–metabolome axis, involving systemic and intestinal metabolic networks, and potentially affecting brain function and excitability (Figure 2). Although gut microbiota alterations have been reported in epilepsy, the mechanisms driving these changes remain incompletely understood. Several factors may contribute to acquired epilepsy-associated dysbiosis, including brain injury- and seizure-induced stress responses, alterations in autonomic nervous system activity (e.g., the enteric nervous system dysfunction and vagal signaling), activation of neuroendocrine (e.g., the hypothalamic–pituitary–adrenal axis) and neuroimmune pathways, and impaired intestinal barrier function. These processes are likely to interact, contributing to the bidirectional communication between brain pathology and the gut microbiota. Elucidating the relative contribution of these mechanisms will be essential for understanding how acquired epilepsy reshapes the gut microbiota and for identifying novel microbiota-targeted therapeutic strategies.

Changes in microbial composition or host metabolism appear to converge on a limited number of interconnected biochemical pathways, including SCFA production, vitamin and cofactor metabolism, amino acid turnover, and tryptophan metabolism. Microbial-derived metabolites emerge as potential functional mediators linking gut dysbiosis to brain dysfunction. In particular, SCFAs represent central regulators of host energy metabolism, neuroimmune signaling, and epigenetic control, whereas alterations in vitamin B6 availability may influence neurotransmitter synthesis and excitatory–inhibitory balance. In parallel, tryptophan metabolism occupies a critical position at the intersection of microbial and host metabolism, with its downstream pathways—serotonin, kynurenine, and indole derivatives—exerting distinct effects on neuronal excitability, neuroinflammation, and blood–brain barrier integrity. Importantly, emerging data suggest that epilepsy-related metabolic alterations are not confined to systemic circulation but are particularly pronounced within the intestinal compartment, highlighting the gut as a crucial site of metabolic reprogramming. This observation reinforces the view that fecal metabolomic profiling may provide a more direct readout of microbiota-driven functional changes compared with plasma-based analyses.

Despite substantial progress, several key questions remain unresolved. It is still unclear whether metabolic alterations observed in peripheral compartments causally contribute to epileptogenesis or represent secondary consequences of disease progression. Moreover, the extent to which intestinal metabolic changes are transmitted to the brain requires further investigation. Addressing these issues will be essential to determine whether microbiota-derived metabolites can serve not only as biomarkers but also as therapeutic targets for epilepsy.

From a translational perspective, SCFA supplementation and other microbiota-targeted interventions are particularly attractive given their favorable safety profile and the increasing clinical evidence supporting their use in neurological and neuropsychiatric disorders. SCFA-based approaches, including butyrate supplementation and related formulations, have been investigated in early-phase clinical studies and are generally well tolerated, supporting their potential suitability as adjunctive therapies to antiseizure medications. Similarly, microbiota-modulating strategies such as probiotic administration have shown encouraging, albeit still largely proof-of-concept, results in small clinical cohorts of patients with epilepsy, suggesting potential benefits on seizure frequency and quality of life. Overall, these findings support the feasibility of translating microbiota–metabolite-based interventions into adjunctive therapeutic strategies for epilepsy, although larger randomized controlled trials are still required to establish efficacy and define optimal treatment regimens.

## Data Availability

The datasets generated and analyzed in the studies by A.V., R.D.S., A.B., T.R. ([15], doi:10.1016/j.bbi.2024.04.007; [16], doi:10.1016/j.nbd.2024.106469; [17], doi:10.1002/ana.78283) are available in the Zenodo repository at https://zenodo.org/records/10795240 (accessed on 7 March 2024) [15]; https://zenodo.org/records/10419539 (accessed on 21 December 2023) [16], https://zenodo.org/records/18683362 (accessed on 18 February 2026) [17]. The data underlying Figure 1C–M are available from the corresponding authors upon reasonable request.
